# Prognosis of synchronous colorectal carcinoma compared to solitary colorectal carcinoma: a matched pair analysis

**DOI:** 10.1097/MEG.0000000000001487

**Published:** 2019-08-06

**Authors:** Wanbin He, Chengjun Zheng, Yonghong Wang, Jie Dan, Mingjie Zhu, Mingtian Wei, Jian Wang, Ziqiang Wang

**Affiliations:** a Department of Gastrointestinal Surgery, The People’s Hospital of Leshan, Leshan; b Department of Gastrointestinal Surgery, West China Hospital, Sichuan University, Chengdu, China

**Keywords:** prognosis, solitary colorectal carcinoma, synchronous colorectal carcinoma

## Abstract

Synchronous colorectal carcinoma (CRC) is a specific and rare type of colorectal malignancy. The data on the impact of synchronous CRC are controversial. This study aimed to compare the characteristics and prognosis between synchronous CRC and solitary CRC.

**Patients and methods:**

252 patients who underwent surgery between October 2009 and June 2013 with synchronous CRC (n = 126) or solitary CRC (n = 126) were included. The patients were matched according to age, sex, American Society of Anesthesiologists score, BMI, cancer grade, tumor location, and tumor stage. The short-term outcomes included the length of hospital stay, complications, and 30-day mortality. Long-term endpoints were overall survival (OS), disease-free survival (DFS), and cancer-specific survival (CSS).

**Results:**

The median follow-up duration for all patients were 42.5 months. The incidence of synchronous CRC was high than in older and male patients as well as in mucinous adenocarcinoma containing signet-ring cell carcinoma, tumor deposit, and polypus. The length of hospital stay after surgery was longer for synchronous CRC than solitary CRC (median: 10 vs. 4 days, *P* = 0.033). In multivariate analysis, synchronous CRC was an independent prognostic factor associated with poor OS (hazard ratio: 2.355, 95% confidence interval: 1.322–4.195, *P* = 0.004), DFS (hazard ratio: 2.079, 95% confidence interval: 1.261–3.429, *P* = 0.004), and CSS (hazard ratio: 2.429, 95% confidence interval: 1.313–4.493, *P* = 0.005).

**Conclusion:**

The clinical and pathological features exhibit differences between synchronous CRC and solitary CRC and the prognosis of patients with synchronous CRC was poorer than those with solitary CRC.

## Introduction

Colorectal cancer (CRC) is the fourth most common carcinoma with an increasing incidence in China [1]. In addition, comparing to solitary tumors, synchronous colorectal carcinoma (synchronous CRC) is a rare type of colorectal malignancy, which is defined as more than one primary colorectal carcinoma detected in one patient at the time of initial presentation [2]. The range of prevalence is 1.1%–8.1% of all colorectal cancers [3]. Synchronous CRC is often seen in males, and a previous large-scale study determined the male/female ratio as 1.85 [4]. The other known higher risk factors include inflammatory bowel diseases, Lynch syndrome, familial adenomatous polyposis, and adenomas/hyperplasic polyposis [5–8]. Moreover, the chromosomal instability, microsatellite instability (MSI), and gene methylation account for various predisposing lesions or factors for synchronous CRC [9].

Although synchronous CRC is identified as a significant entity at the clinical and molecular level, the clinical and pathological features and prognosis are yet controversial [5]. In addition, the results of previous studies which evaluated the prognostic significance of synchronous CRC are conflicting. Thus, the common consensus is reached on the occurrence of synchronous CRC as an independent predictive factor of survival as compared to solitary CRC after the operation [5,10,11]; the expected long-term survival rates of patients with synchronous CRC are yet controversial [12].

The present study aimed to compare the various clinicopathological features and short-term/long-term cancer-specific outcomes between synchronous and solitary CRC by a matched pair analysis with stratification based on age, sex, American Society of Anesthesiologists (ASA) class, BMI, cancer grade, tumor location, and tumor stage.

## Methods

### Patients

This study was approved by the Ethics Committee of West China Hospital of Sichuan University, and the need for informed consent was waived. The study was evaluated by the STROBE statement [13]. A retrospective review of 5742 patients who underwent surgery for colorectal cancer between October 2009 and June 2013 obtained from the database of the Department of Gastrointestinal Surgery of West China Hospital of Sichuan University. A total of 131 (2.8%) patients were diagnosed with synchronous CRC at the time of initial presentation. Of these, five patients who received only enterostomy owing to the tumors did not achieve an R0 reaction, and hence, were excluded from this study. Finally, 126 patients who underwent curative surgery were included in the present study. All the patients accepted colonoscopy. Computed tomography (CT) or magnetic resonance imaging (MRI) was implied to evaluate the resectability before the surgery.

### Matched pair analysis

Each patient with synchronous CRC and a control group of patients with solitary CRC who underwent radical surgery of colon cancer or total mesorectal excision were matched in pairs at a ratio of 1:1. The case-matched criteria included age (± 5 years), sex, ASA class, BMI (± 5 kg/m^2^), cancer grade, tumor location, and tumor stage. In this study, the time of surgery was not considered as a criterion as some patients could not be contacted for follow-up. Thus, the operation time of the control group was restricted to the same period as that of the synchronous CRC group, and the location of the tumor was only matched by two sites: colon and rectum. The pathological stage was determined according to the 7th edition of the American Joint Committee on Cancer staging manual [14]. Matching cancer stage of synchronous CRC was categorized according to the most advanced tumor, and the N-stage was determined based on all lymph nodes.

### Treatment

Either laparoscopic resection or conversion to open surgery was applied by colorectal surgeons who were experienced in colorectal and laparoscopic or conventional surgery. Patients with colon cancer underwent right-sided resections (including the transverse colon), left non-sigmoid resections (including the left colon flexure and the descending colon), or sigmoid resections. The resections of the rectosigmoid junction included a part of the rectum and total mesorectal excision was performed on patients with rectal cancer. Side-to-end anastomosis was used to construct the stoma in the right-sided resections, while straight anastomosis was used in the other resections. All the operations followed the principle: lymphadenectomy and circumferential margins were cleared.

### Assessment parameters

The short-term outcomes included the length of hospital stay, complications, and 30-day mortality. Long-term endpoints were overall survival (OS), disease-free survival (DFS), and cancer-specific survival (CSS). OS was defined as the period from the date of surgery to the date of death from any cause. DFS was defined as the period from the date of surgery to the date of tumor recurrence or distant metastasis. CCS was defined as the period from the date of surgery to the date of death from cancer. The local recurrence was defined as the recurrent disease in the pelvis, while distant recurrence was defined as the recurrence outside of the pelvis. The patients acquired a peritoneal or pelvic sepsis postoperatively, which should be treated by a second operation that was defined as anastomotic leakage.

### Follow-up

All patients were followed-up every 6 months in the initial 3 years and every 12 months thereafter. The clinical evaluations included a complete blood count, liver and kidney function test, serum carcinoembryonic antigen (CEA), carbohydrate antigen 19–9 levels, physical examination (conducted at each visit), CT scan of the abdomen, chest, and pelvis, and colonoscopy (conducted every 12 months). In addition, MRI of the upper abdomen was performed when the patients were suspected with liver metastasis.

### Statistical analysis

The categorical variables were presented as absolute and relative frequencies which were analyzed by the chi-squared or Fisher’s exact probability test. Quantitative variables were reported as mean ± SD and compared by the nonparametric Mann–Whitney *U* test. Odds ratios (ORs) and 95% confidence intervals (CIs) were used as standard measures to quantify the strength of the association between exposure and outcome. The survival curves were calculated using the Kaplan–Meier method and statistical significance were determined by the log-rank test. Univariate and multivariate analyses were conducted using Cox proportional hazards models with the putative clinicopathological parameters included in this analysis. Hazard ratios (HRs) and 95% CIs were used as common measures to assess the relative risk. All statistical analyses were performed using the Statistical Package for the Social Sciences (SPSS), version 22.0 (IBM Co., Armonk, New York, USA). A probability (*P*)-value < 0.05 was considered statistically significant.

## Results

### Demographic data

The present study included 252 (66.7%) patients containing 84 females and 168 males. The mean age of the patients at the time of diagnosis was 62.57 ± 12.63 years for all patients, 62.57 ± 12.64 years for synchronous CRC, and 62.55 ± 12.27 years for solitary CRC. The mean age of the males and females at the time of diagnosis was 63.18 ± 12.73 and 61.36 ± 12.51 years, respectively in synchronous CRC patients.

### Clinical and pathological characteristics

The clinical and pathological characteristics of both groups are listed in Tables 1 and 2, respectively. No significant difference was detected in the age, BMI, sex, ASA score, CEA levels, adjuvant chemoradiotherapy, hospital stay, 30-day mortality, and postoperative complications between the two groups (Table 1). One patient (0.8%) died in each group because of serious pyemia and respiratory failure. Five patients (3.9%) in the synchronous CRC group and three patients (2.4%) in the solitary CRC group developed anastomotic leakage after the operation. The distribution of smoking history (47/126 vs. 28/126, OR: 2.082, 95% CI: 1.197–3.623, *P* = 0.009), alcohol intake (38/126 vs. 18/126, OR: 2.591, 95% CI: 1.383–4.852, *P* = 0.002), and operative approach (103/126 vs. 45/126, *P* < 0.001) differed significantly between two groups. However, the hospital stay after the operation of synchronous CRC was longer than that in solitary CRC (median: 10 vs. 4 days, *P* = 0.033).

**Table 1.**
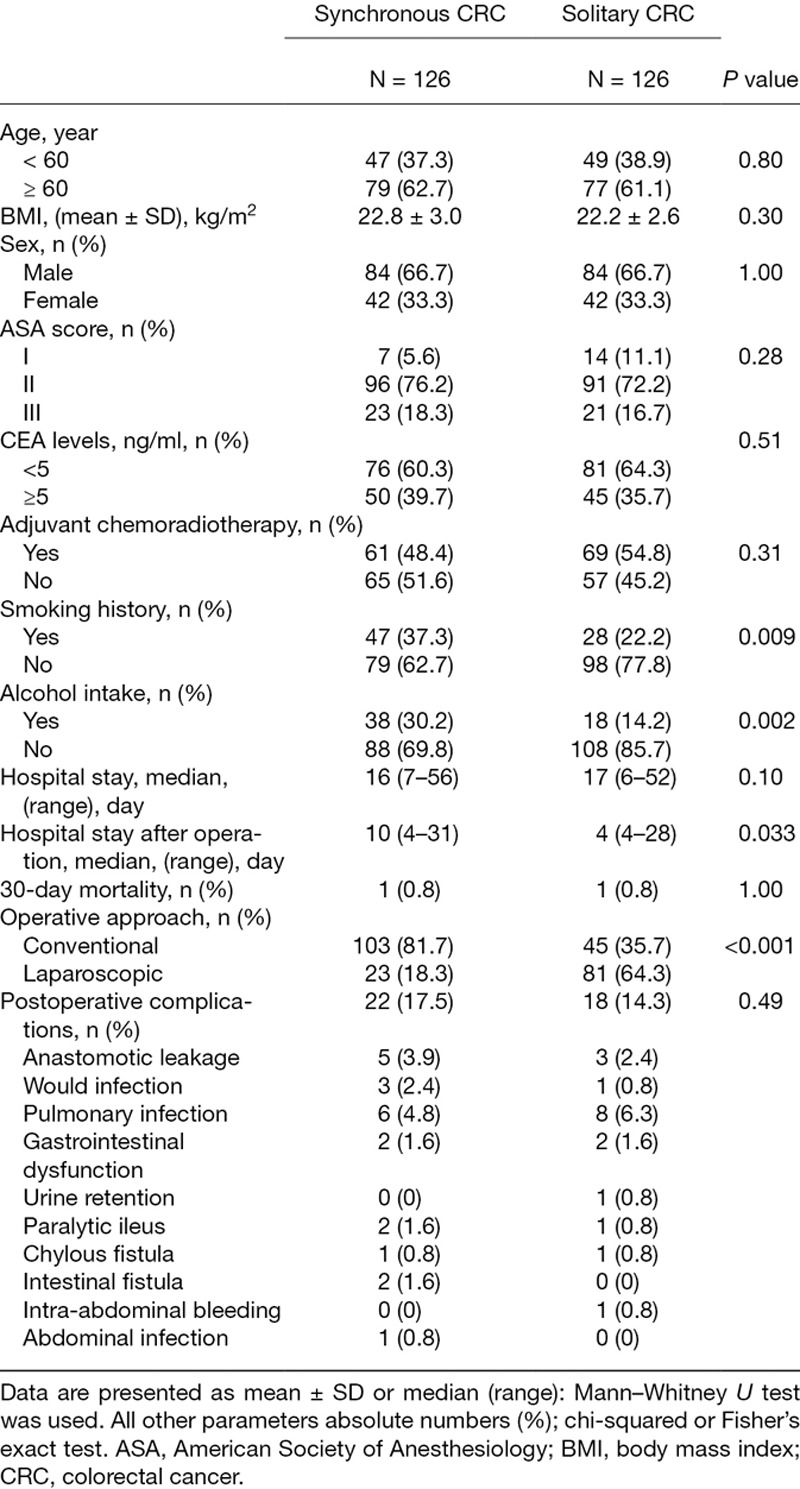
Patient characteristics

Furthermore, no significant differences were found between the two groups with respect to tumor size, tumor location, cancer grade, depth of tumor invasion, regional lymph node status, cancer stage, cancer embolus, and perineural invasion (Table 2). The distribution of mucinous adenocarcinoma (MAC) containing signet-ring cell carcinoma (SC) (50/126 vs. 27/126, OR: 2.412, 95% CI: 1.384–4.204, *P* = 0.002), lymph nodes (78/126 vs. 55/126, OR: 2.098, 95% CI: 1.268–3.470, *P* = 0.004), polypus (57/126 vs. 11/126, OR: 8.636, 95% CI: 4.241–17.586, *P* < 0.001), and tumor deposit (25/126 vs. 13/126, OR: 2.152, 95% CI: 1.045–4.429, *P* = 0.035) was significantly different between the two groups.

**Table 2.**
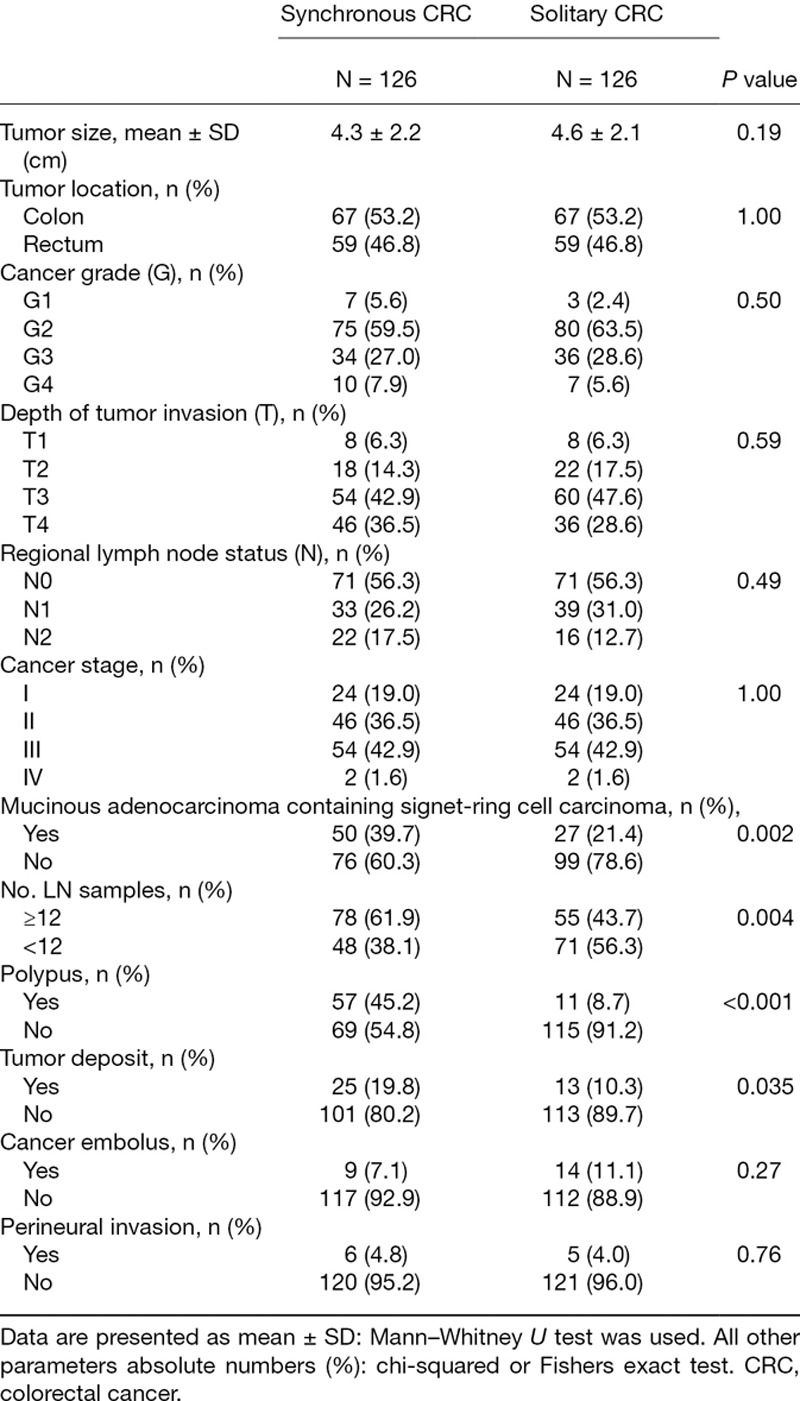
Pathological parameter

In 38 (30.2%) patients, both tumors were localized in the colon, and in 41 (32.5%) patients, both tumors were detected in the rectum. A total of 47 (37.3%) patients had two tumors localized separately in colon and rectum (Table 3).

**Table 3.**
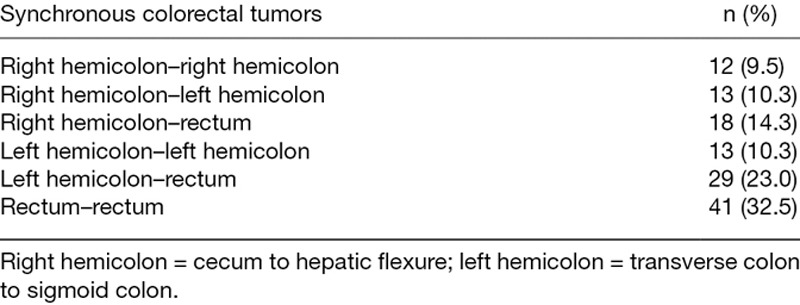
Distribution of synchronous colorectal tumors

### Survival analysis

**Fig. 1.**
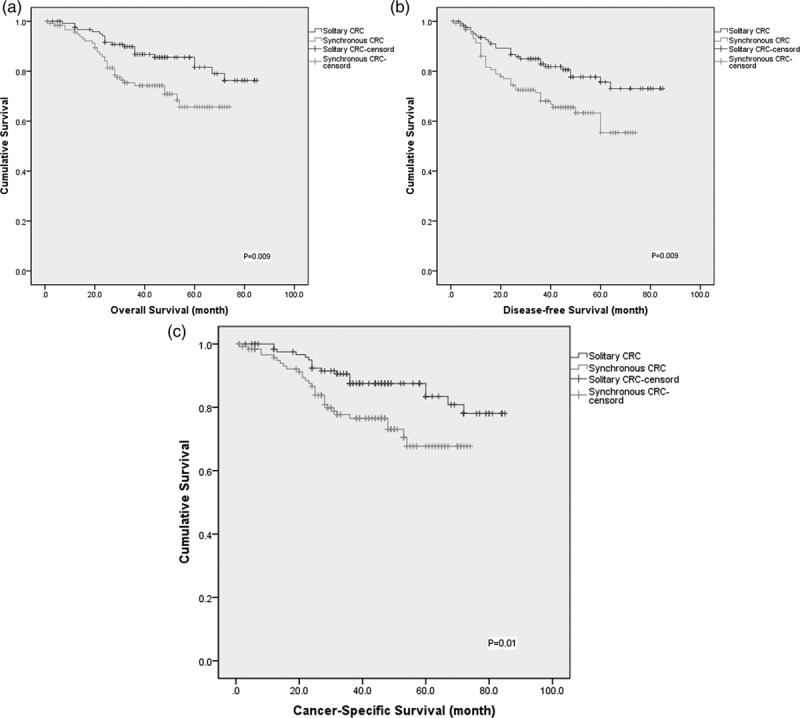
(a) Kaplan–Meier curves of OS for synchronous CRC and solitary CRC patients. (b) Kaplan–Meier curves of DFS for synchronous CRC and solitary CRC patients. (c) Kaplan–Meier curves of CSS for synchronous CRC and solitary CRC patients. CRC, colorectal cancer; CSS, cancer-specific survival; DFS, disease-free survival; OS, overall survival.

The median follow-up time of all patients was 42.5 (range, 1–85) months, while that for synchronous CRC and solitary CRC was 38 (range, 1–74) month and 46 (range, 1–85) months, respectively. The Kaplan–Meier survival curves for OS, DFS, and CSS of the two groups are shown in Fig. 1a, b, and c. The 3- and 5-year OS rates in synchronous CRC group were 74.3 ± 4.2% and 65.7 ± 5.6% and those in the solitary CRC group were 86.8 ± 3.2% and 81.6 ± 4.2%, respectively (*P* = 0.009). The 3- and 5-year DFS rates in synchronous CRC group were 68.1 ± 4.5% and 55.4 ± 6.8% and those in the solitary CRC group were 82.9 ± 3.5% and 75.7 ± 4.5%, respectively (*P* = 0.009). The 3- and 5-year CSS rates in synchronous CRC group were 76.5 ± 4.1% and 67.7 ± 5.6% and those in the solitary CRC group were 87.5 ± 3.1% and 83.5± 4.1%, respectively (*P* = 0.01).

### Univariate and multivariate analysis

The results of the univariate analysis showed that age, sex, ASA score, location of tumor, MAC containing SC, lymph nodes, polypus, cancer embolus, postoperative complications, tumor size, smoking history, and alcohol intake were not associated with the OS, DFS, and CSS (Table 4). CEA levels (HR: 2.141, 95% CI: 1.240–3.679, *P* = 0.006; HR: 1.868, 95% CI: 1.150–3.035, *P* = 0.012; HR: 2.271, 95% CI: 1.278–4.037, *P* = 0.005), cancer grade (HR: 3.296, 95% CI: 1.902–5.711, *P* < 0.001; HR: 2.444, 95% CI: 1.505–3.970, *P* < 0.001; HR: 3.239, 95% CI: 1.818–5.770, *P* < 0.001), regional lymph node status (HR: 4.956, 95% CI: 2.678–9.173, *P* < 0.001; HR: 4.226, 95% CI: 2.492–7.169, *P* < 0.001; HR: 4.331, 95% CI: 2.311–8.117, *P* < 0.001), cancer stage (HR: 4.704, 95% CI: 2.543–8.704, *P* < 0.001; HR: 4.008, 95% CI: 2.363–6.797, *P* < 0.001; HR: 4.109, 95% CI: 2.193–7.698, *P* < 0.001), tumor deposit (HR: 4.095, 95% CI: 2.261–7.417, *P* < 0.001; HR: 3.624, 95% CI: 2.097–6.263, *P* < 0.001; HR: 4.816, 95% CI: 2.618–8.859, *P* < 0.001), perineural invasion (HR: 4.308, 95% CI: 1.529–12.137, *P* = 0.006; HR: 3.488, 95% CI: 1.386–8.781, *P* = 0.008; HR: 3.608, 95% CI: 1.103–11.808, *P* = 0.034), and synchronous CRC (HR: 2.098, 95% CI: 1.190–3.697, *P* = 0.010; HR: 1.906, 95% CI: 1.161–3.129, *P* = 0.011; HR: 2.141, 95% CI: 1.178–3.829, *P* = 0.013) were associated with OS, DFS, and CSS, respectively. On the other hand, the depth of tumor invasion (HR: 2.304, 95% CI: 1.037–5.119, *P* = 0.041; HR: 2.025, 95% CI: 1.031–3.978, *P* = 0.040) was associated with OS and DFS, respectively, and the adjuvant chemoradiotherapy (HR: 2.052, 95% CI: 1.111–3.790, *P* = 0.022) was associated with CSS.

**Table 4.**
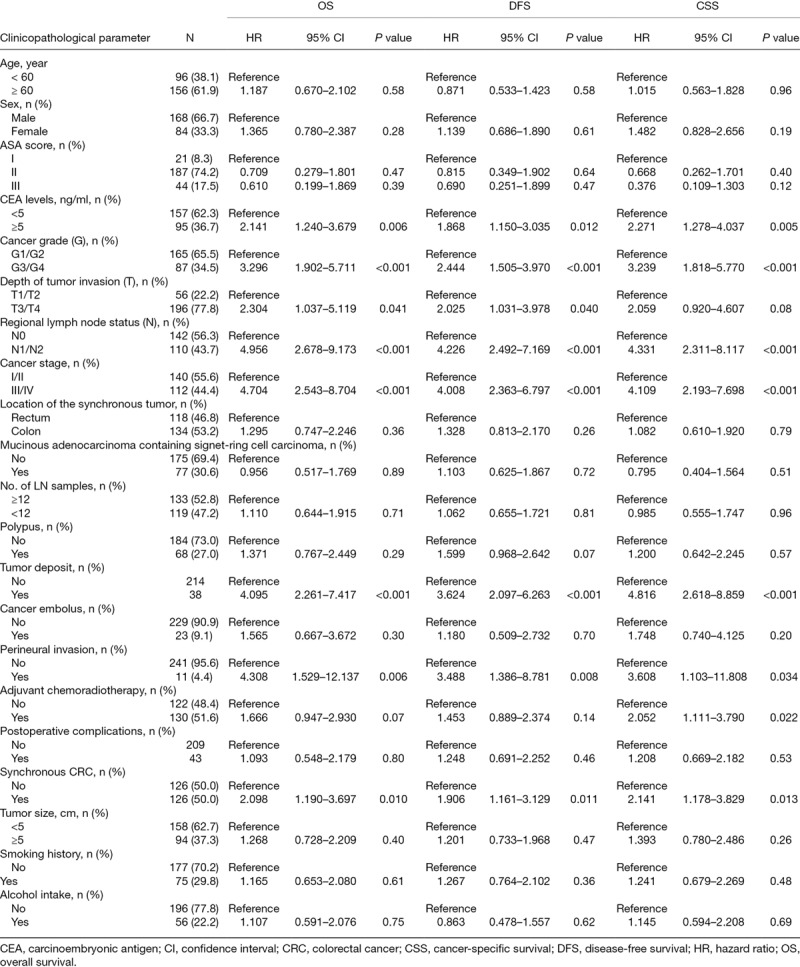
Univariate analysis of clinicopathological parameters for the prediction of overall survival, disease-free survival, and cancer-specific survival

Multivariate analysis was performed to assess the prognostic value of factors with *P* < 0.05 in univariate analysis for OS, DFS, and CSS (Table 5). Cancer grade (HR: 3.336, 95% CI: 1.907–5.835, *P* < 0.001; HR: 2.434, 95% CI: 1.488–3.984, *P* < 0.001; HR: 2.879, 95% CI: 1.574–5.267, *P* = 0.001), regional lymph node status (HR: 4.231, 95% CI: 2.264–7.907, *P* < 0.001; HR: 3.778, 95% CI: 2.209–6.460, *P* < 0.001; HR: 2.854, 95% CI: 1.418–5.747, *P* = 0.003), CEA levels ≥ 5 ng/ml (HR: 1.912, 95% CI: 1.095–3.340, *P* = 0.023; HR: 1.659, 95% CI: 1.012–2.720, *P* = 0.045; HR: 2.017, 95% CI: 1.122–3.624, *P* = 0.019), and synchronous CRC (HR: 2.355, 95% CI: 1.322–4.195, *P* = 0.004; HR: 2.079, 95% CI: 1.261–3.429, *P* = 0.004; HR: 2.429, 95% CI: 1.313–4.493, *P* = 0.005) were independent prognostic factors of OS, DFS, and CSS, respectively.

**Table 5.**
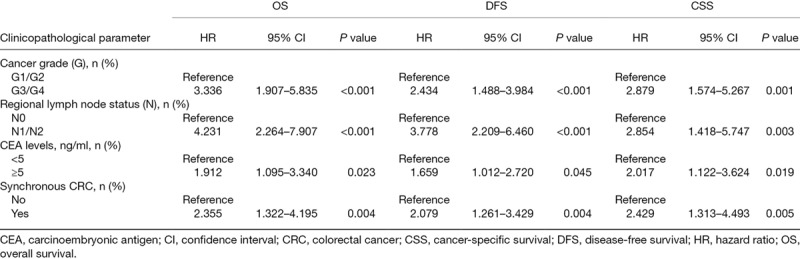
Multivariate analysis of clinicopathological parameters for the prediction of overall survival, disease-free survival, and cancer-specific survival

## Discussion

The present study compared the prognosis of patients with synchronous and solitary in a matched pair analysis. The synchronous CRC, which accounted for 2.8% of all colorectal carcinomas, was an independent prognostic factor for CRC. Comparing to solitary CRC, patients with synchronous CRC had worse long-term OS, DFS, and CSS rates. Cancer grade and regional lymph node status were also correlated with poor OS, DFS, and CSS, while the CEA levels (≥ 5 ng/ml) were associated with poor OS and CSS.

Lam *et al.* [3] reported an overall incidence of 3.5% (3667/105686) of synchronous CRC based on the data from 39 studies. The prevalence of synchronous CRC was < 3.5% in the current study. This phenomenon can be explained by the fact that non-uniform definition and interchanging of synchronous CRC and metachronous CRC [15]. On the other hand, the higher risk of inflammatory bowel diseases was much less common in Asia than in the West, and a lower overall prevalence rate of 2.2% UC-related CRC as compared to the 3%–5% in Western countries [16]. Lynch syndrome also accounted for 5.6%–6.4% of all CRC patients in China [17].

Furthermore, the mean of diagnosis was 62.57 years, which was in concordance with that reported previously [3], and the mean age of the males was higher than that of females (63.18 vs. 61.36 years). With respect to sex, Foster’s study demonstrated a robust association between estrogen activity and metabolism with CRC [18], indicating that the proportion of females with synchronous CRC was less than the males. The risk factors of CRC including family history of colorectal cancer, smoking, excessive alcohol consumption, high consumption of red and processed meat, obesity, and diabetes [19], may also be associated with synchronous CRC. In the current study, smoking history (OR: 2.082, 95% CI: 1.197–3.623) and alcohol intake (OR: 2.591, 95% CI: 1.383–4.852) may lead to a higher risk in patients with synchronous CRC as compared to those with solitary CRC.

Surgery was challenging for synchronous CRC because the tumors were distantly localized. Although colonoscopy can detect and effectively remove the promalignant and malignant lesions [20], it not used commonly in patients with CRC or synchronous CRC. Thus, extensive bowel resection such as total or subtotal colectomy was required in such cases [11]. A previous study reported that patients with synchronous CRC underwent an extensive resection in some cases [15]. In 60 (47.6%) patients, the tumors were localized in different sites, and 103 (81.7%) patients with synchronous CRC accepted open surgery and hospital stay after the surgery duration was longer than that for solitary CRC (median 10 vs. 4 days), which indicated that extended surgical resection is often required in synchronous CRC.

The short-term outcome did not differ significantly between the two groups. The 30-day postoperative mortality was 0.8% in both groups, and no difference was noted in the hospital stay (median 16 vs. 17 days). Five (3.9%) patients with synchronous CRC and three (2.4%) patients with solitary CRC developed anastomotic leakage. Prolonged surgery was correlated with high intra- and postoperative complications and 1.53–9.9 OR for developing anastomotic leakage [21]. However, the long-term outcomes showed a significant difference between the two groups. In multivariate analysis, synchronous CRC was an independent prognostic factor of OS (HR: 2.355, 95% CI: 1.322–4.195, *P* = 0.004), DFS (HR: 2.079, 95% CI: 1.261–3.429, *P* = 0.004), and CSS (HR: 2.429, 95% CI: 1.313–4.493, *P* = 0.005). This difference might be caused by pathological characteristics. In the present study, MAC containing SC in synchronous CRC were more than in solitary CRC (50 vs. 27, OR: 2.412, 95% CI: 1.384–4.204). Another large cohort study demonstrated that MAC and SC were diagnosed at higher tumor stages and associated with a higher risk of tumor recurrence that in turn, reduced the rate of survival [22]. Moreover, the tumor deposit of synchronous CRC was significant in solitary CRC (25 vs. 13, OR: 2.152, 95% CI: 1.045–4.429). The data of SEER also indicated that tumor deposit and perineural invasion correlated independently with poor OS and CSS [23].

The prevalence of MSI-H and mismatch repair (MMR) protein-deficient tumors for synchronous CRC has been controversial. Hu *et al*. [10] reported that the MSI-H CRC accounts for approximately 35% of synchronous CRCs. Nakano *et al*. [24] stated that the frequency of MMR protein deficiency in synchronous CRC in the Japanese population may be lower as compared to that in the Western populations, and MMR protein loss and KRAS and BRAF mutations in synchronous CRCs were heterogeneous in the same patient. However, the prognostic relevance of MSI status of patients with synchronous CRC was yet unclear.

Nevertheless, the present study had some limitations. First, it was a single-center and retrospective design, which might weaken the statistical power of our findings. Additionally, the operation time was not matched accurately, necessitating a prolonged follow-up duration for solitary CRC than synchronous CRC, and hence, the selection bias could not be excluded.

## Conclusion

Synchronous CRC is a unique subtype of colorectal cancer with marked disparity in clinical and pathological implications as compared to solitary CRC. The results of the present study showed a similar short-term outcome of synchronous CRC and solitary CRC patients in a matched pair analysis; however, patients with synchronous CRC exhibited worse OS, DFS, and CSS than those with solitary CRC.

## Acknowledgements

We thank all the patients who participated in this study and provided the follow-up data. This work was supported by the Science and Technology Support Program of the Science and Technology Department of Sichuan Province (No. 2016SZ0043). This study was approved by the Ethics Committee of West China Hospital of Sichuan University, and the need to obtain informed consent was waived. Conceived and designed the experiments: Z.Q.W. and J.W. Data collection: W.B.H., M.T.W., and J.D. Analyzed the data: W.B.H., Y.H.W., and M.J.Z. Wrote the paper: W.B.H. and C.J.Z.

## Conflicts of interest

There are no conflicts of interest.
